# 670nm Photobiomodulation as a Novel Protection against Retinopathy of Prematurity: Evidence from Oxygen Induced Retinopathy Models

**DOI:** 10.1371/journal.pone.0072135

**Published:** 2013-08-08

**Authors:** Riccardo Natoli, Krisztina Valter, Marconi Barbosa, Jane Dahlstrom, Matt Rutar, Alison Kent, Jan Provis

**Affiliations:** 1 The John Curtin School of Medical Research, Australian National University, Canberra, Australian Capital Territory, Australia; 2 ARC Centre of Excellence in Vision Science, Australian National University, Canberra, Australian Capital Territory, Australia; 3 ANU Medical School, Australian National University, Canberra, Australian Capital Territory, Australia; 4 Department of Anatomical Pathology, Canberra Hospital, Woden, Australian Capital Territory, Australia; 5 Department of Neonatology, Canberra Hospital, Woden, Australian Capital Territory, Australia; Massachusetts Eye & Ear Infirmary, Harvard Medical School, United States of America

## Abstract

**Introduction:**

To investigate the validity of using 670nm red light as a preventative treatment for Retinopathy of Prematurity in two animal models of oxygen-induced retinopathy (OIR).

**Materials and Methods:**

During and post exposure to hyperoxia, C57BL/6J mice or Sprague-Dawley rats were exposed to 670nm light for 3 minutes a day (9J/cm^2^). Whole mounted retinas were investigated for evidence of vascular abnormalities, while sections of neural retina were used to quantify levels of cell death using the TUNEL technique. Organs were removed, weighed and independent histopathology examination performed.

**Results:**

670nm light reduced neovascularisation, vaso-obliteration and abnormal peripheral branching patterns of retinal vessels in OIR. The neural retina was also protected against OIR by 670nm light exposure. OIR-exposed animals had severe lung pathology, including haemorrhage and oedema, that was significantly reduced in 670nm+OIR light-exposed animals. There were no significance differences in the organ weights of animals in the 670nm light-exposed animals, and no adverse effects of exposure to 670nm light were detected.

**Discussion:**

Low levels of exposure to 670nm light protects against OIR and lung damage associated with exposure to high levels of oxygen, and may prove to be a non-invasive and inexpensive preventative treatment for ROP and chronic lung disease associated with prematurity.

## Introduction

Retinopathy of Prematurity (ROP) is a disorder of the developing retina, and one of the leading cause of blindness in infants of the western world, contributing heavily to the social and financial burdens caused by visual impairment worldwide [1]. There are many risk factors leading to progression of ROP including low birth weight, gestational age, supplemental oxygen therapy, sepsis, blood transfusions, respiratory distress syndrome and genetics [2–5]. However gestational age, low-birth weight and supplemental oxygen are the widely accepted major risk factors.

ROP has been described as a two phase disease, beginning with delayed vascular growth after premature birth (Phase I – hyperoxic phase), followed by the release of hypoxia stimulated factors to stimulate new blood vessel growth (Phase II – hypoxic phase) [6]. Supplemental oxygen is administered to premature infants to maintain adequate oxygenation levels, to overcome pulmonary insufficiency. Since being identified as a risk factor for ROP, supplemental oxygen is now monitored closely leading to a decrease in the number of infants developing ROP. However, as the survival rates of very low birth weight infants (VLBW) increase, so does the incidence of ROP [7]. A number of clinical studies are underway to determine the safe oxygen saturation levels at which premature infants can be nursed to minimise the risk of ROP [8] and as a result, a number of guidelines have been developed for screening and treatment of ROP in premature and low birth weight infants [9]. Use of these guidelines significantly reduces the risk of blindness and significant visual loss, but does not prevent the disease from occurring, nor the other long-term visual consequences of ROP.

Current therapies for ROP, including laser photocoagulation or cryo-therapy to reduce the incidence of blindness, although a number of long-term visual issues can still present post treatment including reduced visual acuity [10], reduced visual fields [11], reduced contrast sensitivity [12] and strabismus [13]. All current treatments for ROP are invasive, expensive and target only the angiogenic aspect of the disease, thus not addressing the neuronal impacts or other long-term effects. Thus there is a need for an inexpensive, non-invasive preventative treatment for ROP.

Low-level Light therapy (LLLT) in the red to near-infrared light spectrum (600-1000nm) protects against neuronal and retinal cell damage [14], and 670nm red light has been shown to be a powerful neuroprotectant, against light induced damage [15,16] and toxins [17]. Treatment with 670nm red light improves retinal healing [18] and modulates expression of genes involved with inflammation, oxidative metabolism and apoptosis [19]. Although the precise mechanism is unknown there is strong evidence to suggest that cytochrome c oxidase (CCO) acts as the primary photo-acceptor/chromophore [20], boosting oxidative metabolism [21] and ATP production [22], driving reparative and protective mechanisms.

Animal models for ROP are well established and include a number of rodent models. These oxygen-induced retinopathy (OIR) models have provided crucial insights into the pathogenesis and underlying mechanism of ROP [23]. OIR models take advantage of the fact that in some animals normal retinal vascularisation occurs *ex utero*, and thus resemble the incomplete vascular development of the retinal vasculature in premature infants.

The aims of the present study were to use mouse and rat models to evaluate the efficacy of 670nm red light to protect against OIR. We hypothesise that treatment with 670nm red light will be a novel preventative strategy for ROP, promoting normal mechanisms of retinal vascularisation, reducing oxidative and neuronal damage.

## Materials and Methods

### Animals

All work was conducted using either C57BL/6J mice or Sprague-Dawley albino rats. All animal experimentation was conducted in accordance to the ARVO (Association for Research in Vision and Ophthalmology) statement for the Use of Animals in Ophthalmic and Vision Research and with the approval of the Animal Ethics Committee at the Australian National University, Canberra (protocol-A2011/029). Animals were raised and experiments conducted in cyclic 5 lux light (12hrs: 12hrs). All animals were culled using cervical dislocation. All culling was performed at 9am to control for possible circadian effects.

For both the mouse and rat OIR models animals were assigned to one of 4 groups; control (normal oxygen, no 670nm), 670nm (normal oxygen, 670nm treatment only), OIR (oxygen only, no 670nm treatment) or 670nm+OIR (oxygen and 670nm treatment). To adjust for litter size and cross-litter variability all litters were maintained at 10 animals per experimental group and 2 experimental groups per cage/dam (control and 670nm, or OIR and 670nm + OIR). All experiments were performed in biological triplicate (3 litters per experimental group), with a minimum of 12 animals per experimental group.

### Oxygen Induced Retinopathy Models

#### Mouse 75% oxygen model

Animals were maintained in normoxia until P7. At P7 dam and pups were placed in an Oxycycler (Biospherix Ltd., Lacona, NY) with constant 24hrs 75% oxygen for 5 days. At p12 animals were removed from oxygen and returned to normoxia until P17. At P17 animals were culled and the eyes and organs harvested for further investigation. Animal weights and lengths were measured daily at 9am. Animals weighing less than 4g at P17 were excluded from the study.

#### Rat 80%/21% oxygen model

At birth (P0) pups and dam were placed in an Oxycycler (Biospherix Ltd., Lacona, NY) with 24 hours cyclic oxygen, 80% for 22 hours: 21% (normoxia) for 2 hours. Animals were maintained this way until P18 when they were culled, and the eyes and organs harvested for further investigation. Animal weights and lengths were measured daily. Animals weighing less than 30g at P18 were excluded from the study.

### 670nm Red Light Treatment

Treated animals were exposed to 670nm red light only during exposure to hyperoxia (P7-P17, mouse; P0 – P18, rat). Animals were exposed to 670nm red light from a WARP 75 source (Quantum Devices Inc., Barneveld, WI) Each animal was held approximately 2.5cm from the light source and treated for 3 minutes daily. This arrangement provided a fluence of 9J/cm^2^ at the eye. The animals did not appear agitated by the red light. All treatments were performed at 9am.

### Retinal Whole-mounts

Retinal whole-mounts were prepared using an established technique [24] with slight modifications. Eyes were removed and immediately fixed in 4% paraformaldehyde for 1 hour. Following fixation the eyes were rinsed 3 times in phosphate buffered saline (PBS). The retinas were removed from the eyecup, and placed under a dissection microscope (Leica, Wetzlar, Germany). To whole-mount the retina an ophthalmic surgical blade was used to create four incisions 1mm from the optic nerve head to the peripheral retina. Retinas were fixed in 4% paraformaldehyde for four hours then washed overnight in PBS. Retinas were then washed with PBS for three intervals of 30 minutes and placed in a fluorescein isothiocyanate (FITC) conjugated lectin stain (Sigma, St. Louis, MO) at a ratio of 1:100 with PBS for 24 hours, followed by rinsing with PBS for three intervals of 30 minutes. Retinas were then whole-mounted on slides and visualised using LSM 5 confocal microscope (Carl Zeiss, Jena, Germany) and acquired using PASCAL v 4.0 software (Carl Zeiss). Individual images were stitched together to create whole retinal images and prepared for publication using Adobe Photoshop CS4. Assessment of the neovascularisation and vaso-obliteration was performed as a blinded assessment using a technique described previously [24]. To quantify vaso-obliteration, lectin images were imported into Adobe Photoshop, the avascular area selected and the pixel density quantified. The number of pixels for the vaso-obliteration was normalised against the total number of pixels of the whole mount, giving a percentage of vaso-obliteration. Neovascularisation was calculated using a macro in imageJ [25] designed to specifically select areas of neo-vascularisation. The number of pixels for the neovascularisation was normalised against the total number of pixels of the whole mount, giving a percentage of neovascularisation. A one-way ANOVA with Tuckey’s *post hoc* test was performed using Prism 5 (GraphPad Software) to compare the effects of different treatment conditions.

### Peripheral Branching

We used a recently submitted image processing method (Barbosa and Maddess, manuscript in preparation) namely Streamlined Morphological Image Analysis (SMIA) to characterize the change in vessel arborisation due to different treatments or conditions. SMIA features in 2D are related to known geometric measures such as the Area, Perimeter and the Euler number of a region. These quantities are monitored while the region of interest is undergoing a variational segmentation process called Chan-Vese [CV]. At critical points of this process, the most rich shape representation of the object occurs and we extract the features for SMIA, automatically.

Having the SMIA geometrical features at hand we performed a one way analysis of variance (MANOVA) using their principal components in order to quantify the distance between the treatment groups. We display the result hierarchically by clustering the resulting Mahalanobis distance using a single linkage (nearest neighbour) algorithm.

### Organ Weights and Pathology

Following cervical dislocation, animals organs were removed, weighed fresh then fixed in 10% neutral buffered formalin (NBF) and analysed for any histological abnormalities. The organs removed included the lungs, brain, kidney, liver, spleen, heart and thymus. Pathology was assessed blind and performed by an anatomical pathologist experienced in rodent histology at the Canberra Hospital, Australia.

### Cryosectioning and TUNEL

In some animals, the fellow eye was marked on the superior aspect with a pen for orientation, enucleated and immersion-fixed in 4% paraformaldehyde for 3 hours. The eye was then washed in PBS three times and cryoprotected by immersion in 15% sucrose overnight. Eyes were sectioned at 12µm on a cryostat in the superior-inferior axis.

Cell death was assessed by the TdT-mediated dUTP nick end labelling (TUNEL) technique to identify the fragmentation of DNA characteristic of apoptotic cells, following a previously published protocol [26] but using a fluorophore, Alexa 594 for visualisation. TUNEL-labelled sections were scanned from superior to inferior edge and the number of TUNEL+ profiles in each of the nuclear layers of the retina was recorded. Frequency of TUNEL+ profiles/mm was averaged from at least three sections per animal with 4 animals analysed. A one-way ANOVA with Tuckey’s *post hoc* test was performed using Prism 5 (GraphPad Software) to compare the effects of different treatment conditions.

## Results

### Effects of 670nm on Vascular Development in Oxygen Induced Retinopathy

**Figure 1:**
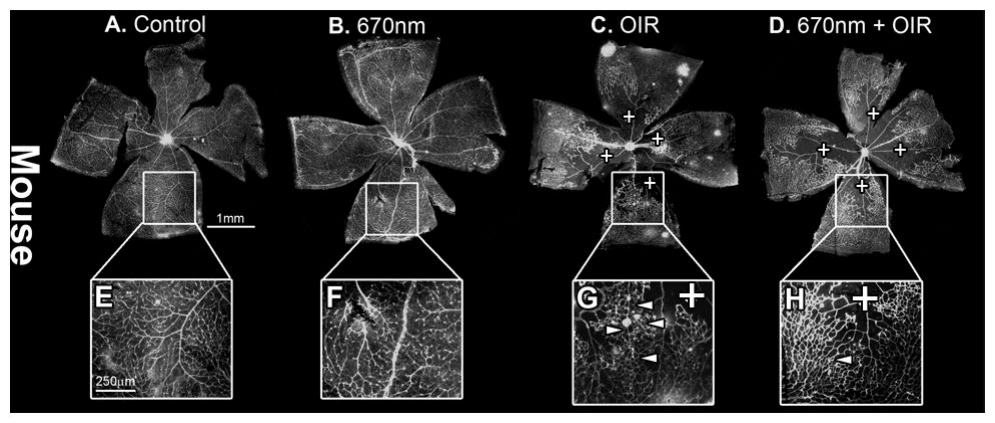
P17 c57BL/6J mice retinal wholemounts stained with lectin. (A,E) Control and (B,F) 670nm alone mice retina showed no observable difference in peripheral retinal vasculature, while (C,G) OIR alone showed increaed vaso-obliteration (indicated by **+**) and neovascularisation (indicated by arrowheads). There was also an observable vascular organisational difffernce in the OIR exposed retina (G). (D, H) 670nm+OIR, showed amelioration of neovascularisation, vaso-obliteration and a more uniform vascular patterning. P – postnatal day, OIR - Oxygen induced retinopathy. Scale A - D = 1mm, E -H = 200µm, I. Magnification of images E-H are 4x original images.

**Figure 2:**
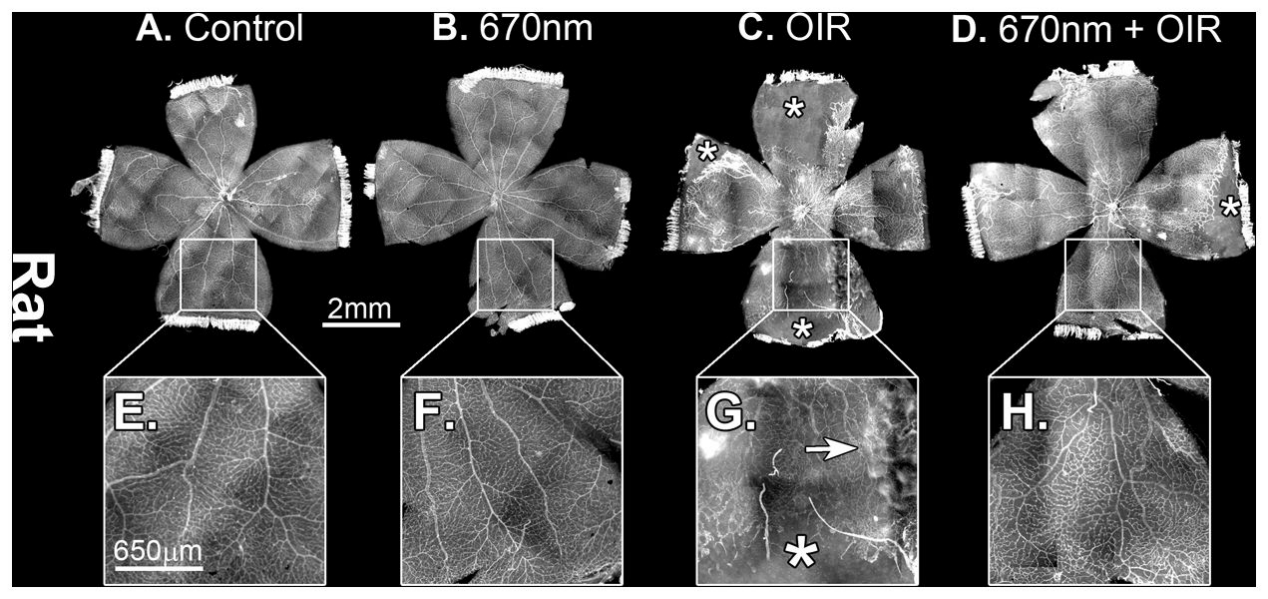
P18 Sprauge-Dawley rat retinal wholemounts, stained with lectin. (A,E) Control and (B,F) 670nm alone mice retina showed no observable difference in peripheral retinal vasculature, while (C,G) OIR alone showed increaed vaso-obliteration (indicated by **+**) and areas of retinal heamorrhages (indicated by an arrow). (D,H) 670nm+OIR showed amelioration of vaso-obliteration and control like vascular patterning. P – postnatal day, OIR - Oxygen induced retinopathy. Scale A-D = 2mm, E-H = 650µm. Magnification of images E-H are 4x original images.

**Figure 3:**
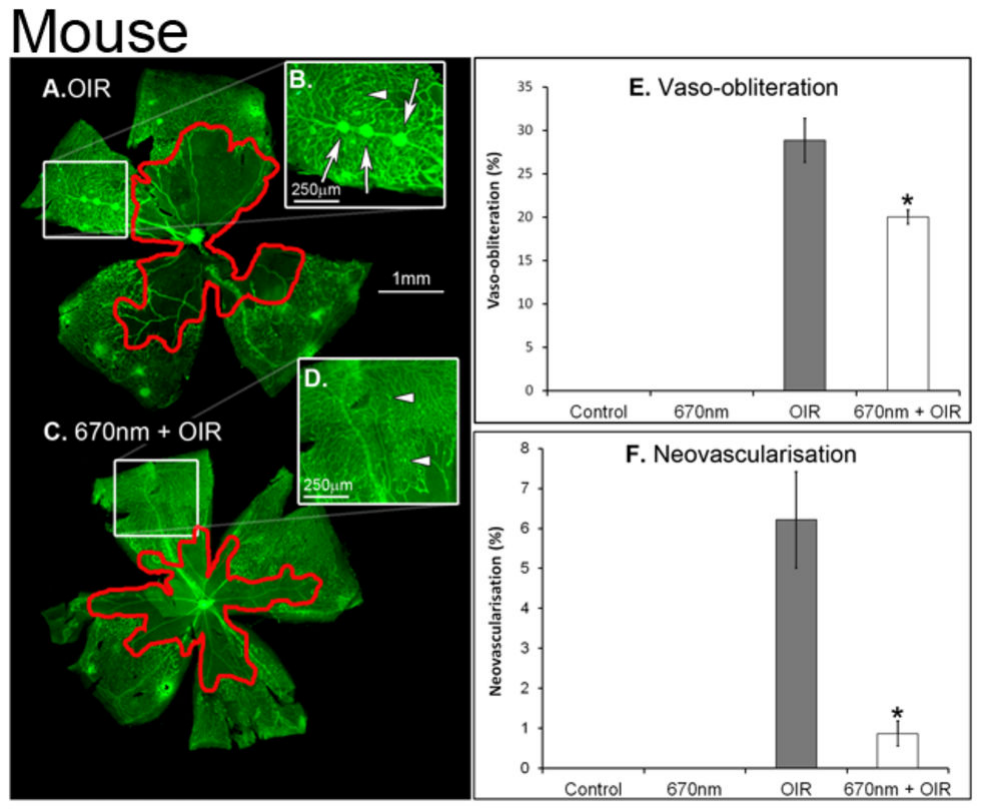
Quantification of vascular abnormalities in P17 c57BL/6J mouse lectin stained retinal wholmounts. (A, B) OIR shows increased vaso-obliteration (E) and neovascularisation (F) compared to 670nm + OIR (C, D). Neovascularisation was reduced to almost control levels in the 670nm+OIR retina (F). Red (A, C) areas show vaso-obliteration, while white arrows show large neovascular tuffs and white arrows show small tufts. * indicated statistical significance p<0.05. Error bars show standard error. P – postnatal day, OIR – Oxygen induced retinopathy. Graph bar fills indicate controls (black), 670nm treated (dark grey), OIR (light grey) and 670nm+OIR (white).

**Figure 4:**
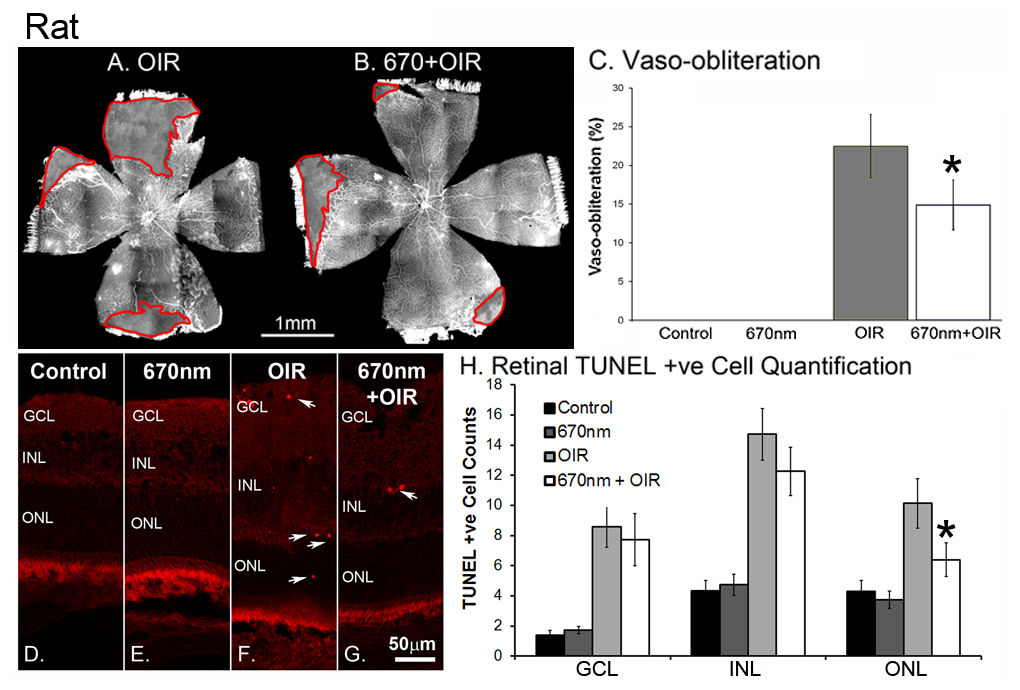
Quantification of vaso-obliteration and cell death in P18 Sprague-Dawley rats. (A) OIR shows increased vaso-obliteration (C) compared to 670nm + OIR (B). Red (A, B) areas show vaso-obliteration. Representative images (D–E) and quantification (H) of TUNEL images from the rat retina. To maintain consistency representative images (D–E) were all taken 500µm from the optic nerve head on the superior side of the retina. Arrows indicate positive cell labelling in both the OIR and 670nm+OIR animals. Control and 670nm (dark showed little TUNEL positive labelling (H) in any of the retinal layers, while experimental groups exposed to OIR showed increased labelling compared to control levels (H). 670nm+OIR reduced the level of labelling from that of OIR, but only with statistical significance in the ONL (H). * indicated statistical significance p <0.05. Error bars show standard error. Arrows indicate positive cell labelling. GCL – ganglion cell layer, INL – Inner nuclear layer, ONL – outer nuclear layer, P – postnatal day, OIR - Oxygen Induced Retinopathy. Scale A,B = 1mm, D-G = 50µm. Graph bar fills indicate controls (black), 670nm treated (dark grey), OIR (light grey) and 670nm+OIR (white).

Retinal whole-mounts from mice (Figure 1) and rats (Figure 2) revealed the modifications of retinal vascular development typical of OIR paradigms, including vaso-obliteration, neovascularisation and retinal haemorrhages. In the mouse model vaso-obliteration occurs centrally, while in the rat model it occurs on the retinal periphery. In both control and ‘NIR alone’ animals, there was no adverse vascular development (n=12). Quantification of both vaso-obliteration (Figure 3 A, C, E and Figure 4 A–C) and neovascularisation (Figure 2 B, D and F) showed that in mice retinas treated with 670nm there was a significant decrease in the severity of vascular pathology (n=12, p value < 0.05). In the rat vaso-obliteration was reduced from 4.1% in OIR to 3.2% in OIR+670nm treated animals (Figure 4C). In mice vaso-obliteration was reduced from 28.9% in OIR to 20.0% (Figure 3E) in OIR+670nm animals, while neovascularisation was reduced from 6.2% to 0.9% (Figure 3F). In mice the amount of retinal neovascularisation in OIR retinas treated with 670nm was reduced to almost that of control (Figure 3F) (n=12, p value = 0.0128).

**Figure 5:**
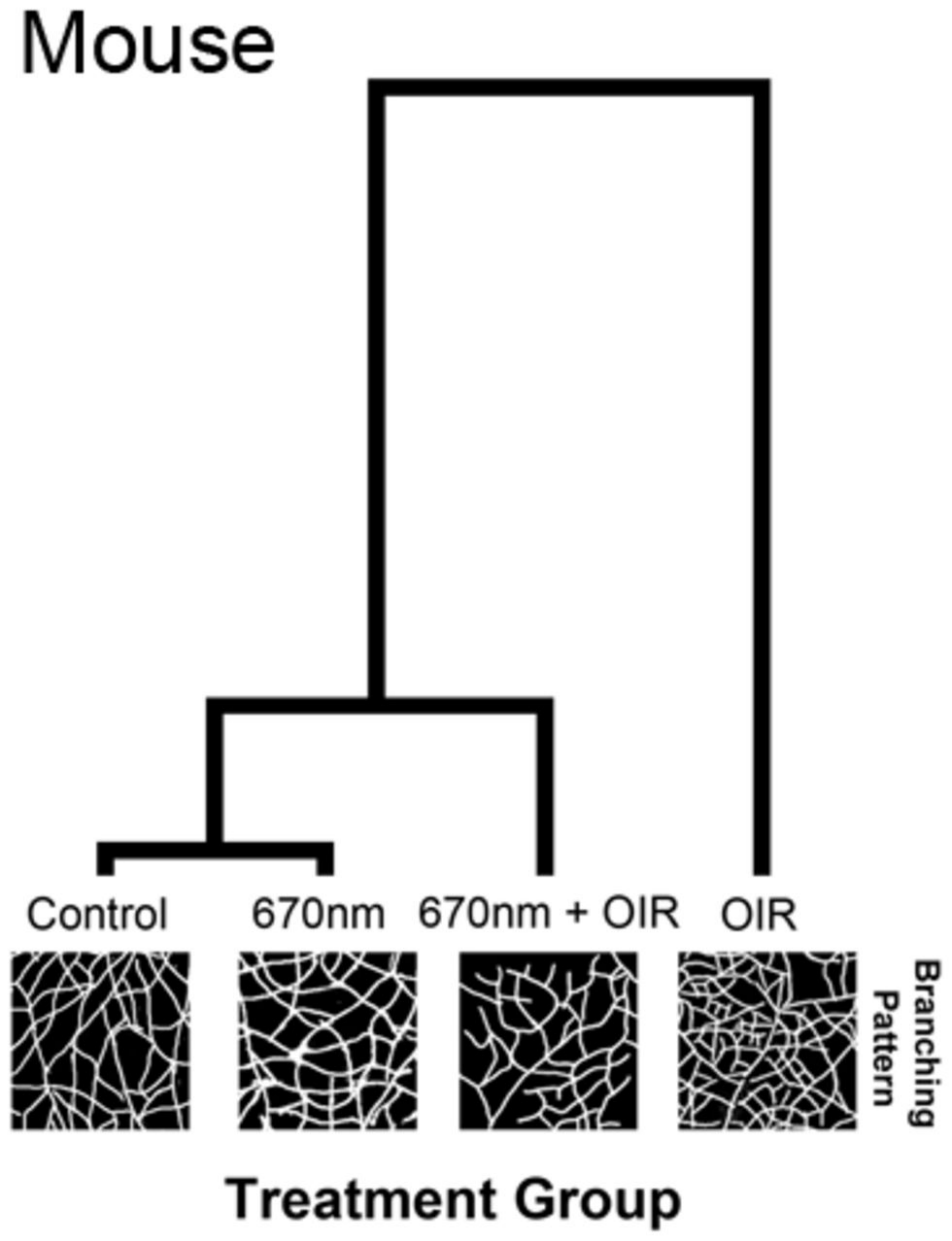
Analysis of peripheral vessel branching patterns in the mouse OIR model. The hierarchical clustering diagram indicates a divergence from the experimental groups with the largest divergence occurring with the OIR group. The Mahalanobis distance was calculated from a MANOVA procedure and is shown relative to control. The OIR group clusters alone (1.96 distance) indicate a very different pattern of peripheral vasculature, to the other experimental groups. 670nm+OIR (0.46 distance) reduced the alteration in the peripheral patterning to almost that of control and 670nm (0.32 distance). The length of the lines in the hierarchical clustering diagram indicate the relative difference between groups.

**Figure 6:**
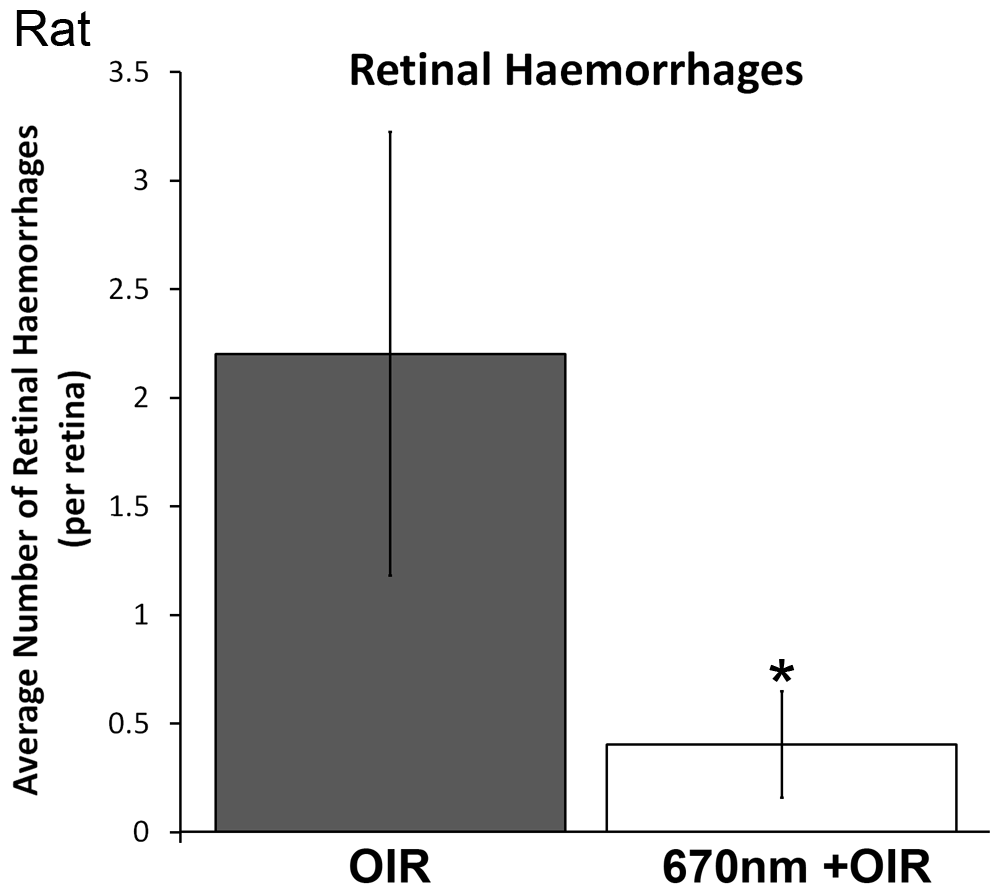
Number of retinal haemorrhages in the rat oxygen induced retinopathy animals. The number of haemorrhages was reduced from 2.3 per retina in the OIR animals to 0.4 in the 670nm+OIR animals. The 670nm+OIR was not statistically significant from controls, but was from OIR animals (* indicates a p-value < 0.05). OIR – Oxygen Induced Retinopathy. Graph bar fills indicate OIR (light grey) and 670nm+OIR (white).

The peripheral retinal vascular branching patterns in the OIR-exposed animals were anomalous, including increased tortuosity and disorganization in the peripheral vessel network (Figure 1C,G, Figure 2C,G). Analysis of the retinal peripheral vasculature branching patterns showed that OIR retinas had a distinctive pattern of branching, compared to controls. The cladistic analysis (Figure 5) indicates that OIR retinas clustered separately from the other three experimental groups, and that 670nm+OIR retinas have a peripheral branching pattern that is closely related to that seen in controls and 670nm. Furthermore, quantification of the number of rat retinal haemorrhages (Figure 6) shows that rats subjected to the OIR paradigm, but also treated with 670nm light had relatively few retinal haemorrhages, statistically different from OIR alone (n=12, p value = 0.0135).

### Effects of 670nm on Photoreceptor Cell Death in Oxygen Induced Retinopathy

Natural cell death is a normal feature of retinal development and, as expected, there were TUNEL positive cells present in control and ‘NIR only’ samples; these values were not statistically different from each other (n=12, p value > 0.05). Analysis of retinal cell death (rat model) showed a significant increase in the overall amount of cell death in retinas exposed to the OIR paradigm (Figure 4 D–H) (n=12, p values < 0.05). The data also show that retina from 670nm+OIR animals had reduced levels of cell death in all retinal layers, compared with OIR alone (Figure 4H), however only in the outer nuclear layer (ONL) was this found to be statistically significant (n=12, p = 0.018). This indicates that 670nm was able to protect against OIR in the ONL layer of the retina.

### Effects of 670nm on Growth and Organ Development in Mice Raised in Normoxia

**Figure 7:**
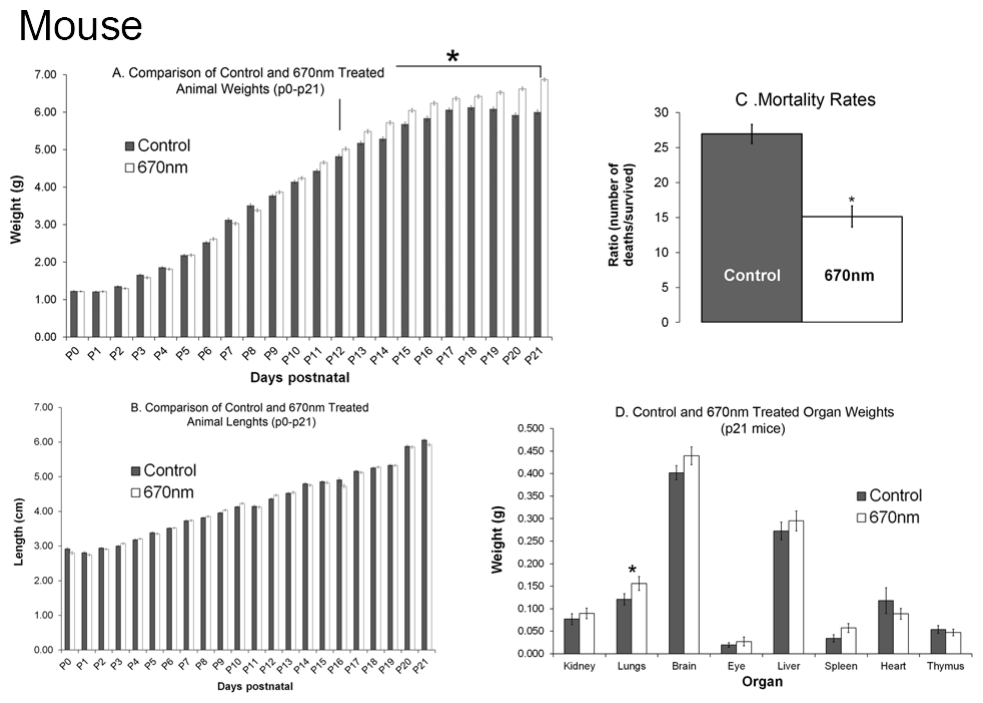
Animal weight (A), length (B), mortality rate (C) and organ weight (D) taken daily in animals exposed to 670nm compared to controls in normal atmospheric oxygen. (A) From P12 there was a statistically significant (* indicated p<0.05) deviation from normal weight gain with animals gaining weight at an increased rate. (B) There were no noticeable differences in the length of the animals over the 21 day period. (C) There was no change in the weight of the organs investigated except for the lungs which showed a significant increase in weight (* indicates p < 0.05). (D) 670nm red light appears to reduce the mortality rate from 27% in controls (n=27) to 15% in the 670nm (n=21) exposed animals (* indicates p<0.05). All animals were raised in normal atmospheric oxygen. Littermates were used to reduce variability across litters. Graph bar fills indicate controls (grey bars), 670nm treated (white bars).

No changes in length were noticed in the animals (control n=27, treated n=21) during 21 days of monitoring (Figure 7B). However, we detected an increase in the weight of the treated animals between P12 – P21 days (Figure 7A; p value < 0.05) which was no longer evident in animals allowed to develop to adulthood. Analysis of organ weight (control n=27, treated n=21) showed no difference in the 670nm treated animals (Figure 7D), except for the lungs which were significantly heavier in the 670nm treated animals (p value < 0.05). Histopathological analysis of the organs showed no difference between the experimental groups. We also noted that in the group of mice treated with 670nm light there was a reduced mortality rate; 27% in controls, 15% in 670nm treated mice (Figure 7C). In animals allowed to develop to adulthood following 670nm red light treatment there were no adverse findings in organs, or overall size of the animals.

### Organ Development in Oxygen Induced Retinopathy

In normal development of the control rats, eyes were open by P14 in 100% of animals (n=24). In rats exposed to the OIR paradigm eye-opening was delayed, such that only 50% of animals had opened their eyes by P15, and 100% at P16 (n=12). Eye opening occurred earlier in groups treated with 670nm light, such that 60% of rats treated with 670nm alone (n=12) opened eyes by P13, and 25% of 670nm+OIR (n=12) had open eyes by P14. Rats exposed to the OIR paradigm had reduced weight and length compared to controls; exposure to 670nm light did not modify this. There was no effect of 670nm light treatment alone on normal animal weight and length.

**Figure 8:**
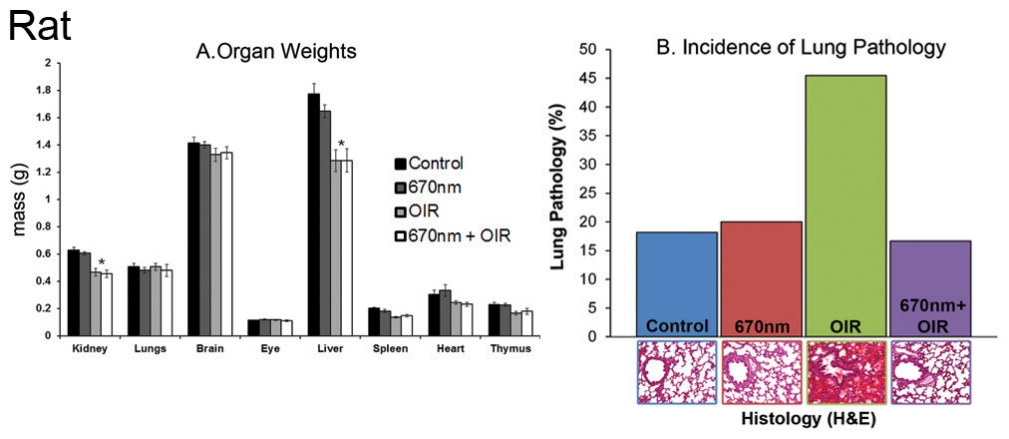
Assessment of the organ weights and lung pathology of rats exposed to either OIR, 670nm or both. There was no change in the weights of animals exposed to 670nm from control (A). The OIR animals showed a statistically significant decrease in organ weight in both the kidneys and liver (* indicates p <0.05). There was an increase in the number of lung pathology (B) presentations (both haemorrhages and oedema) in the OIR exposed animals. This was ameliorated by 670nm exposure compared to control levels. 670nm alone showed no change from control levels. 670nm treated OIR animals did not show any difference from OIR levels. OIR – Oxygen Induced Retinopathy. Graph bar fills indicate controls (black), 670nm treated (dark grey), OIR (light grey) and 670nm+OIR (white).

Both the kidneys and livers of rats exposed to the OIR paradigm were reduced in size compared to controls (Figure 8A) (p < 0.05), although histopathological analysis showed no differences in most of the organs. An exception was the lungs; rats exposed to the OIR paradigm had increased numbers of haemorrhage and areas of oedema compared to controls. Significantly, lung pathology in 670nm+OIR rats were consistent with control (Figure 8B).

## Discussion

This is the first study to demonstrate that 670nm red light therapy of neonatal animals can modify the adverse outcomes of exposure to high levels of oxygen and protects the retina from OIR. Furthermore, the data show that in rats treatment with 670nm red light for short exposures (3 minutes a day) for 18 days does not induce pathology in major organs or any alterations in normal development. When administered in conjunction with an OIR paradigm, the present study shows that in both the mouse OIR paradigm, and the rat OIR paradigm, 670nm red light treatment (i) reduces vaso-obliteration in the retinal vasculature; (ii) reduces neovascularization and retinal haemorrhage; (iii) preserves retinal vascular branching architecture, and (iv) reduces the incidence of neural cell death. Furthermore, we find that treatment of OIR rats with 670nm red light reduces the incidence of lung pathology to levels that are comparable with controls.

The animal models used in this study are established models for mimicking the sort of pathologies seen in ROP (reviewed in 23), with strengths and weaknesses to both models. The differences between the mouse and rat OIR models include differences in the patterns of vaso-obliteration, such that in the mouse OIR model vaso-obliterations occur centrally, around the optic disc [27], while in rats the vaso-obliteration occurs in the periphery [28], as they do in human infants. The mouse model, however, is better suited to quantifying changes in levels of neovascularisation [27]. The retina of albino neonates (reviewed in 29 also respond differently to fluctuations of oxygen [30] and have developmental mutations, such as the inability to regulate retinal cell cycle [31]. Despite these differences we observed a similar and significant reduction in the severity of vaso-obliteration in both rat and mouse OIR animals treated with 670nm red light.

ROP involves vaso-obliteration and neovascularisation, and in the worst cases results in neural damage, retinal detachment and blindness [6,32]. The hyperoxic phase of OIR results from inspired oxygen being administered to neonates in order to maintain normal oxygen saturations in infants affected by lung disease of prematurity. Although, inspired oxygen therapy does not account for all occurrences of ROP, it is widely accepted as one of the key factors in the pathogenesis of the disease [2,33]. This makes the OIR models useful for studying changes in retinal pathology due to exposure to increased (mouse) or fluctuating (rat) oxygen levels. Our results suggest that the severity of the hypoxic phase of ROP can be reduced, by maintaining a normal vessel development during the hyperoxic phase, thus reducing the impact of hypoxia on return to room air.

It is unclear how normal development of the retinal vasculature is maintained by 670nm red light in these models of OIR. This is largely because the precise mechanism underling the effect of LLLT is largely unknown. The most widely accepted hypothesis is that cytochrome c oxidase is the photoacceptor for 670nm red light [20,22,34,35], and promotes an increase in oxidative metabolism (reviewed in 36). Thus it is suggested that 670nm red light acts to promote mitochondrial function, increasing oxygen usage, reducing oxidative damage, and increasing ATP production [37,38]. This hs been shown in number of non-neuronal cells, such as astrocytes [39] and fibroblast [40] however has been most effective in CNS tissue (reviewed in 36). This is probably due to the high metabolic demands of neuronal tissue, and subsequent susceptibility to damage as a result of fluctuations in oxidative metabolism. In these OIR models it is possible that 670nm red light promotes consumption of the excess oxygen available in the hyperoxic phase of the disease, making oxygen levels in the tissues closer to normoxia, and facilitating more normal development of the retinal vasculature.

Establishing normoxia in the retinal microenvironment implies a moderation of the oxidative stress conditions that result from hyperoxia. Indeed recent experiments show that in adult mice exposed to hyperoxia, levels of acrolein and haemoxygenase, both markers of oxidative stress, are significantly reduced in the retina by exposure to 670nm red light (Albarracin, manuscript in preparation). A reduction in oxidative stress may also explain reduced natural cell death in the retina, and could minimise damage [9] to organs susceptible to fluctuations in oxygen levels, including the lungs. While the present results support this hypothesis, more work is required to understand the mechanism/s of action of 670nm red light. An alternative hypothesis is that neuroglia cells, including astrocytes, which are damaged by OIR experimental conditions [41] are protected by 670nm red light exposure during the hyperoxic phase, preserving normal processes of vascular development. This idea is consistent with previous findings that 670nm red light exposure moderates the expression of inflammatory markers and complement in retinal degeneration induced by bright light [42].

Finally, photoreceptor dysfunction and retinal cell death have been reported in OIR animal models [43] and in ROP [44]. Neuroprotection is therefore an important aspect for evaluation of ROP treatment and management. This study shows that the neural retina, and specifically the photoreceptors, are protected from cell death and OIR by 670nm red light therapy. Such protection of photoreceptors is consistent with other models, including the rodent light damage model [45], indicating that oxidative damage is a common feature in photoreceptor death [15,16,19]. Excessive white light [45] and certain wavelengths of light [46] have been shown to be toxic to photoreceptors, however there is no evidence to suggest 670nm is damaging to photoreceptors [15,16,18,19,42]. This is possibly due to the mechanisms of phototoxicity and photobiomodulation being different with white light acting on the photopigments, leading to lipid peroxidation and oxidative stress [47] and 670nm red light acting on the cytochrome c oxidase promoting efficient mitochondrial function.

Although not the focus of this study our findings indicate that 670nm treatment may be beneficial in the management of other complications of prematurity, in particular Chronic Lung Disease (CLD). Hyperoxic and hypoxic states also influence the development of CLD [48], which in turn contributes to vulnerability to ROP because of the fluctuations in arterial oxygenation related to the lung disease and oxygen treatment required to maintain normal saturations. It is possible that 670nm red light may reduce the severity of CLD, by reducing inflammation and stabilising lung development and alveolarisation during hyperoxia, resulting in more ordered vascular development, and fewer haemorrhages. Systemic changes to inflammation have been described in a recent study [49] where inflammatory markers were modulated following low-level-laser therapy (660nm) treatment in an animal model of heart failure. This study supports these previous findings that systemic changes to biology, and specifically inflammation, can occur from localised LLLT and presents the first indication that 670nm red light can protect the lungs from CLD. Indeed, we suggest that such a decrease in inflammation and increase in metabolic efficiency explains the decreased mortality of mice treated with 670nm red light. However, further work is required to document the influences of 670nm red light on lung development and neonatal survival.

## Conclusion

As modern-medicine pushes the survivability limits of pre-term babies, we must endeavour to find solutions to the complicated problems associated with lower preterm survivability, such as the increased incidence and severity of ROP. Exposure to 670nm red light is a potential novel, non-invasive and inexpensive treatment for the prevention and moderation of ROP.
